# Hippocampal encoding of interoceptive context during fear conditioning

**DOI:** 10.1038/tp.2016.254

**Published:** 2017-01-03

**Authors:** S-W Yoo, M Bae, L B Tovar-y-Romo, N J Haughey

**Affiliations:** 1 Department of Neurology, Richard T. Johnson Division of Neuroimmunology and Neurological Infections, The Johns Hopkins University School of Medicine, Baltimore, MD, USA; 2 Department of Psychiatry, The Johns Hopkins University School of Medicine, Baltimore, MD, USA

## Abstract

Rodent models of auditory fear conditioning are often used to understand the molecular mechanisms regulating fear- and anxiety-related behaviors. Conditioning and extinction memories are influenced by contextual cues, and the reinstatement of conditioned fear occurs when the conditioning stimulus is presented in a context different from the extinction context. Although it has been proposed that internal state is a feature of context that could influence extinction, contributions of interoception to conditioning have not been experimentally addressed. Here we use ethanol (EtOH) to show that interoceptive cues are encoded through the hippocampus by mechanisms that involve increased phosphorylation of GluR1 on serine 845, and biophysical alterations in neuronal membranes that facilitate stabilization of surface-located calcium-permeable n-2-amino-3-(5-methyl-3-oxo-1,2-oxazol-4-yl) propanoic acid (AMPA) receptor (AMPAR) into membrane microdomains. Conflicting interoceptive cues during extinction and fear relapse testing resulted in a failure to consolidate extinction that was reversed by the administration of AMPAR antagonists immediately following the retrieval cue.

## Introduction

Rodent models of auditory fear conditioning have proven to be useful tools for understanding the molecular mechanisms underlying fear-related behaviors including posttraumatic stress disorders (PTSD). Fear conditioning involves the pairing of a conditioned stimulus (CS; tone) with an unconditioned stimulus (US; mild electric shock) to produce a conditioned response (freezing). Conditioned fear is said to occur when repeated CS–US pairing results in the CS, eliciting the conditioned response in the absence of the US. Repetitive CS in the absence of US results in progressive reductions of the conditioned response through a process known as extinction (the rodent equivalent of desensitization). Roles for the medial prefrontal cortex, amygdala and hippocampus in fear conditioning and extinction have been extensively described, with contributions for each structure determined by the circumstances of conditioning.^1, 2, 3, 4^

The process of extinction does not appear to erase the conditioning memory, but is thought to generate a new memory that competes with the conditioning memory.^1^ This extinction memory is less robust than the conditioning memory, and the permanence of extinction is influenced by context and the amount of time that has elapsed since extinction. It is well established that the extinction of fear conditioning is more durable when conducted in a novel environment, and renewal of conditioned fear can occur when the CS is presented outside of the extinction context.^5^ Contextual cues are typically thought to include spatial and sensory components that are encoded through hippocampal circuitry;^6^ however, it has also been proposed that the internal state of an organism could contribute to the perception of context. Roles for this interoception in regulating contextual aspects of extinction have not been experimentally tested. As alcohol impairs both the perception of environmental cues, and internal perceptions (how one feels), we reasoned that alcohol-associated impairment of extinction could involve modifications of internal context. Here we provide evidence that alcohol interferes with behavioral extinction by modifying the perception of context, and that these interoceptive cues are encoded through modifications in the phosphorylation and surface expression of calcium-permeable n-2-amino-3-(5-methyl-3-oxo-1,2-oxazol-4-yl) propanoic acid (AMPA) receptors (CP-AMPAR) in the hippocampus. The effects of conflicting internal context on the permanence of extinction can be rescued when AMPA receptors (AMPAR) are blocked immediately following the retrieval cue, and before extinction training.

## Materials and methods

### Animals

Male C3-C57B1/6 J mice (8–12 weeks of age) were obtained from the Jackson Laboratory (Bar Harbor, ME, USA). Animals were single-housed in a temperature- and humidity-controlled room under a 12-h light cycle with food and water available *ad libitum*, except during self-administration of ethanol (EtOH) when water was withheld. Animals were allowed to acclimate to the colony room for at least 7 days after arrival and all behavioral experiments were carried out during the dark cycle. All animal procedures were approved by the Johns Hopkins University Animal Care and Use Committee.

### EtOH self-administration

Binge drinking of EtOH was conducted using a previously published method^7^ modified from Rhodes.^8^ EtOH (Sigma, St Louis, MO, USA; 200 proof, ⩾99.5%) was diluted to 20% (*v*/*v*) in sterile water and administered in water bottles for 2 h beginning 2 h into the animals' dark cycle. Water bottles were pre-tested to ensure they did not leak, and consumption was determined by water weight before and after administration. The amount of EtOH intake was recorded for each day after the 2 h drinking period. Vehicle controls were given water in the similar containers as mice who received EtOH, and they were handled in same manner as mice exposed to EtOH. Blood EtOH was measured three times during behavioral training: immediately following binge EtOH intake, following extinction training and following fear relapse training using an Ethanol assay (Sigma) that uses a coupled enzyme reaction to oxidize EtOH and measure hydrogen peroxide as a fluorometric product.

### Fear conditioning reconsolidation, recovery and measurement of fearing behavior

Fear conditioning, reconsolidation and fear relapse testing were performed using methods similar to previous reports with slight modifications.^9^ Customized sound isolation boxes containing a modular test cage with an electrifiable floor grid and ambient light supply were used for conditioning (Coulbourn Instruments, Whitehall, PA, USA). On the first day of conditioning (Day 0), mice were presented with six auditory tones (CS) each paired with electric shock (US). Before the fear conditioning, a period of acclimation lasting 240 s preceded the presentation of cues. The CS consisting of an 80-dB 2-KHz pure tone lasting 10 s and the US consisting of scrambled 1.0-mA foot shock lasting 2 s were terminated after the paired conditioning. Following conditioning, mice were returned to their home cages until preparation of brain slices or further behavioral examination. On the day of reconsolidation update (Day 1), mice were divided into two groups as control (normal sterile water) and ethanol (20% in sterile water). Mice were depleted of water for 6 h and then were provided water or ethanol for 2 h at beginning of 2 h into their dark cycle.^8^ Reconsolidation was performed in a context distinct from that in which conditioning took place, and consisted of a textured polymer box with scents removed with 70% ethanol. A single CS was presented during the 30-s retrieval period, and animals were returned to their homecages. Extinction training was conducted within 15 min of reconsolidation and consisted of two different blocks of 18 exposures to the CS alone (80-dB 2-KHz pure tones lasting 10 s) separated by 10-s intervals. After reconsolidation update, mice were returned to their homecages. On day 2, mice were returned to the same chambers that were used for extinction training and presented with four CS to measure fear relapse. Fear behavior measured as the percent time freezing during CS was quantified using an automated motion-sensitive software (Clever system, Reston, VA, USA).

### Drug treatments

Mice were intraperitoneally injected with Talamapanel (5 mg kg^−1^; Medkoo Bioscience, Chapel Hill, NC, USA) once or three times (6 h interval) following fear condition, or once following exposure to the retrieval cue. Perampanel (5 mg kg^−1^, Medchem Express, Monmouth Junction, NJ, USA) was administered in a separate group of mice once following the retrieval cue. Control mice and EtOH-only mice were injected with Vehicle (2.5% dimethylsulphoxide in 0.9% NaCl). Immediately following fear relapse testing, open field testing was conducted to measure locomotion activity following the administration of Talampanel. The open field consisted of a square acrylic box incorporating an automated activity monitor (Cage Rack Flex-Field Photobeam Activity System, San Diego Instruments, San Diego, CA, USA), which provides horizontal and vertical grids of 16 × 16 infrared beams. The total number of beam breaks in both horizontal and vertical planes over a period of 10 min (120 s, five times, averaged) was recorded and analyzed. For direct hippocampal infusions of the CP-AMPAR antagonist Naspm trihydrochloride (Naspm), mice were implanted bilaterally with 26-gauge stainless steel external cannulas (model C235G, Plastics One, Roanoke, VA, USA) into CA1 region of the hippocampus (anterior/posterior, −2.0 mm; medial/lateral, ±1.5 mm; dorsal/ventral, 1.5 mm from bregma) 7 days before fear conditioning. Naspm (4 μg per 0.5 μl per hippocampus) was infused into a 33-gauge internal cannula (model C235I, Plastics One) using a 10 μl Hamilton syringe at a rate of 0.5 μl min^−1^ in freely moving animals. Internal cannulas were left in place for an additional 1 min to allow for drug to diffuse into parenchyma before returning animals to the testing chamber.

### Cell culture and experimental treatments

Hippocampal neuronal cultures were prepared from embryonic day 18 Sprague–Dawley rats using methods that have been described previously.^10^ In brief, hippocampi were isolated, trypsinized and cells were dissociated by gentle trituration in a calcium-free Hank’s balanced salt solution (Gibco, Grand Island, NY, USA). Neurons were plated at a density of 150 000 cells per ml on 15 mm diameter with or without polyethylenimine-coated glass coverslips depending on purpose in Neurobasal media (Gibco) supplemented with B27 (Gibco) and 1% antibiotic solution (10^4^ U ml^−1^ of penicillin, 10 mg ml^−1^ of streptomycin and 25 μg ml^−1^ of amphotericin B (Gibco)). Three hours after plating, media was completely replaced, and thereafter supplemented with Neurobasal media containing B27 every 7 days. Hippocampal cultures are routinely >98% MAP-2+ neurons, with the remainder of cells predominantly GFAP+ astrocytes. Hippocampal cultures were used between 14 and 21 days *in vitro*. Ethanol was diluted from stock concentrations just before experiments and was used at final concentrations ranging from 20 to 100 mM for indicated time.

### Immunofluorescence, confocal microscopy and quantification

Membrane microdomains were identified using a cholera toxin subunit B conjugated to Alexa Fluor 555 (CTB-555; Invitrogen/Molecular Probes, Carlsbad, CA, USA) that binds the ganglioside GM1.^10^ CTB-555 (1 ng ml^−1^) was incubated with neurons for 10 min at 37 °C in a 5% CO_2_ atmosphere. Media was rapidly removed and cells were fixed with an ice-cold 4% paraformaldehyde solution prepared in Tris-buffered saline (TBS, 25 mM Tris, 150 mM NaCl, 2 mM KCl; pH 7.4). Membranes were permeabilized and nonspecific binding was blocked by incubation for 1 h at room temperature in TBS containing 0.1% Triton X-100 and 5% normal goat serum. Cells were then incubated with primary antibodies overnight at 4 °C that included anti-GluR1 antibodies (clone C3T; 1:100, Millipore, Bedford, MA, USA) and GluR2 (clone 6C4, 1:100, Millipore). Slides were washed with TBS and incubated for 2 h at room temperature with secondary antibodies tagged with AlexaFluor 488 (1:1000 dilution; Invitrogen/Molecular Probes). Immunopositive puncta on dendritic branches were imaged with a × 100 objective lens using a Zeiss Axiovert microscope equipped with an ApoTome and Axiocam MRm camera and Axiovision (4.8.2) imaging software (Carl Zeiss, Oberkochen, Germany). For figures, images were deconvoluted with module with Axiovision (4.8.2) imaging software (Carl Zeiss). Quantification of immunofluorescence was conducted using methods similar to those previously described.^10^ All images for quantification were taken with identical settings under the same conditions. For each image the threshold was adjusted manually so that the immune-labeled regions corresponded to puncta with intensities that were at least twofold above the diffuse fluorescence on the dendritic branch. Quantifications were conducted on single-plane images through the brightest point, and criteria for a positive identification were that the puncta must be clear and distinguishable. Lipid raft size was determined by tracing the boarder of each CTB-555-immunopositive region within a defined region of the dendrite. The numbers of GluR1 and GluR2 were determined by counting the number of immunopositive puncta in defined areas of dendrites by an investigator blinded to the experimental condition. Within 100 μm of the soma, the area was calculated for each region of interest by tracing the outline of the dendrites. Calibrations of pixels to μm^2^ were accomplished with the Open Lab software (Improvision; Perk and Elmer, Waltham, MA, USA). Each species of GluR1 or GluR2 was considered to be microdomain-located if there was any pixel overlap between CTB-555 and the secondary antibody AlexaFluor 488. To account for treatment-induced increases in the size of CTB-555+ regions, the number of co-localized puncta were normalized to the interested area for each experiment. A minimum of 21 dendrites in at least three separate cultures were quantified for each experimental condition.

### Isolation of detergent-resistant membrane raft and immunoblotting

Membrane rafts were isolated according to the previous report with slight modification.^11^ Briefly, detergent-resistant membrane rafts were isolated from primary neurons using ice-cold lysis buffer, MBS buffer (25 mM MES, 150 mM NaCl, pH 6.5), containing 10 mM MgCl_2_, 10 mM NaF, 2 mM Na_3_VO_4_; 1 mM EGTA, 0.2 mM phenylmethyl sulphonyl fluoride pH 8.0 and 1.0% *w*/*v* CHAPSO (Sigma) as the detergent. Lysates were incubated on ice for 30 min and sonicated. Following sonication, 1 ml from the total homogenate was mixed with 1 ml of 90% (*w*/*v*) sucrose in MBS buffer and placed in a 5 ml ultracentrifuge tube. A 5-35-45% sucrose gradient was formed by layering 2 ml of 35% sucrose in MBS buffer on top of 2 ml 45% sucrose containing homogenate, followed by 5% (1 ml) sucrose. The gradient was centrifuged at 47 000 r.p.m. for 18 h at 4 °C in a Beckman MLS-50 Swinging bucket rotor (Beckman Coulter, Brea, CA, USA). A light-scattering band located at the 25–30% interface was identified indicating the presence of lipid rafts. Ten 0.5 ml fractions were collected from the top of the ultracentrifuge tube and proteins were analyzed by immunoblotting. For immunoblotting of the fractions, equal volumes of each fraction were resolved by 10% sodium dodecyl sulphate-polyacrylamide gel electrophoresis (SDS-PAGE) and transferred to polyvinylidene difluoride membranes (Bio-Rad, Hercules, CA, USA). Nonspecific binding sites were blocked with 5% (*w*/*v*) milk in TBS containing 0.1% Tween 20 (TBS-T). After blocking, blots were incubated overnight with the primary polyclonal antibody Flotillin 1 (1:1000, Abcam, San Francisco, CA, USA), or monoclonal antibodies GluR1 (clone RH95, 1:1000, Millipore) and transferrin receptor (1:1000, Invitrogen). Following washes with TBS-T, blots were incubated for 2 h with the appropriate IgG horseradish peroxidase-linked antibody (1:1000; Cell Signaling Technology, Danvers, MA, USA), and developed by enhanced chemiluminescence. Image analysis was performed using a G:BOX Imaging system (Syngene, Frederick, MD, USA).

### Calcium imaging

Cytosolic calcium levels ([Ca^2+^]_c_) were measured using the Ca^2+^-specific fluorescent probe Fura-2AM. Rat hippocampal neurons were incubated for 20 min with Fura-2AM (2 μM) at 37 °C in Neurobasal media containing B27 supplement. Neurons were washed with Locke’s buffer (154 mM NaCl, 3.6 mM NaHCO_3_, 5.6 mM KCl, 1 mM MgCl_2_, 5 mM HEPES, 2.3 mM CaCl_2_ and 10 mM glucose; pH 7.4) to remove extracellular Fura-2 and incubated at 37 °C for an additional 10 min to allow complete deesterification of the probe. Coverslips containing Fura-2-loaded cells were mounted in an RC-26 imaging chamber (Warner Instruments, Hamden, CT, USA) and maintained at 37 °C (TC344B Automatic Temperature Controller; Warner Instruments). Neurons were perfused at the rate of 2 ml min^−1^ with Lockes buffer using a V8 channel controller (Warner Instruments). Rapid switching from Lockes buffer to n-2-amino-3-(5-methyl-3-oxo-1,2-oxazol-4-yl) propanoic acid (AMPA; 10 μM, Tocris, Ellisville, MS, USA) containing ethanol or Naspm trihydrochloride (Naspm, 50 μM, Sigma) was accomplished by placing the perfusion tube and suction apparatus close to the cells to be imaged (with an ~0.05 cm gap) so that a thin film of perfusate rapidly passed over the cells. Cells were excited at 340 and 380 nm, and emission was recorded at 510 nm with a video-based intracellular imaging system (Photon Technology, Edison, NJ, USA) equipped with a QuantEM 512-sc electron-multiplying gain camera (Photometrics, Tuscon, AZ, USA). Images were acquired at the rate of 200 ms per image pair from 10 to 15 regions (~1 μM^2^) along dendritic branches. The fluorescent intensities of ratio images were converted to nM [Ca^2+^]_c_ by curve fitting using reference standards as previously described.^10^ Ethanol (60 mM) was pretreated at 2 min before AMPA (20 μM) and retreated with AMPA or washed out depending on the experimental paradigm. To induce surface expression of AMPA by increasing cAMP, 50 μM of 3-isobutyl-1-methylxanthine (Sigma) and 20 μM of forskolin (Tocris, Bristol, UK) were used. For direct comparisons of focal AMPA-evoked calcium responses with the location of GluR1 to lipid raft or non-raft microdomains, cells were fixed following calcium recordings, and immunostained as described with CTB-555 and GluR1.

### Biotinylation of surface receptors

Surface expression of receptors was determined in cultured cells using a biotin-labeling kit (Pierce Chemical, Rockford, IL, USA) as described by the manufacturer. Cerebral cortical cells were cultured on polyethylenimine (PEI)-coated 60 mm dishes for 14 days. On day 15, cells were incubated with ethanol and/or protein kinase A (PKA) and protein kinase C (PKC) modulators, forskolin+3-isobutyl-1-methylxanthine and AMPA or vehicle. The cells were gently mixed with EZ Link Sulfo-NHS-SS-Biotin (0.5 mg ml^−1^, Pierce Chemical) in phosphate-buffered saline (1 mM MgCl_2_, 1 mM CaCl_2_, 137 mM NaCl, 2.7 mM KCl 10 mM Na_2_HPO_4_ and 0.2 mM KH_2_PO_4_) reagent on a rocker for 30 min in 5% CO incubator on ice. The cells were washed twice with ice-cold phosphate-buffered saline containing 192 mM Glycine to remove unbound biotin. The cells were then lysed with 600 μl lysis buffer (50 Mm Tris-HCl, pH 7.5, 150 mM NaCl, 1% Triton X-100 0.5% deoxycholate, 30 mM NaF 1 mM ortho-vanadate and protease inhibitor), scraped and transferred to a tube, sonicated on ice for 10 s and centrifuged at 500 *g* for 5 min. Biotin-labeled proteins and flow-through (cytosolic proteins) were separated with 30 μl 50% NeutrAvidin slurry (Pierce Chemical) as described by the manufacturer. The biotinylated (surface) proteins were eluted from the beads by incubation for overnight at 4 °C and washed twice. An equal volume of SDS-PAGE sample buffer (6.25 mM Tris-HCl, pH 6.8, 15% glycerol, 2% SDS, 1% β-mercaptoethanol, 2 mM dithiothreitol and 0.02% bromophenol blue) at 95 °C 20 min was used. The samples were then subjected to gel electrophoresis and western blotting as described above with primary antibodies recognizing GluR1 (clone C3T; 1:1000, Millipore), GluR2 (clone 6C4, 1:1000, Millipore) and phosphor-GluR1 on serine 831 (clone N453; 1:1000, Millipore). To see specific kinase-dependent phosphorylation of GluR1, PKA inhibitor, KT5720 (Tocris, 1 μM) and PKC inhibitor, chelerythrine (Tocris, 1 μM) were pretreated. All comparisons were made within blots.

### Statistics

All of the results were analyzed using one-way analysis of variance followed by Tukey *post hoc* analyses when group differences were significant. Results are expressed as mean±s.d. or mean±s.e. where indicated.

## Results

### EtOH creates conflicting interoceptive cues that result in a failure to consolidate behavioral extinction

**Figure 1:**
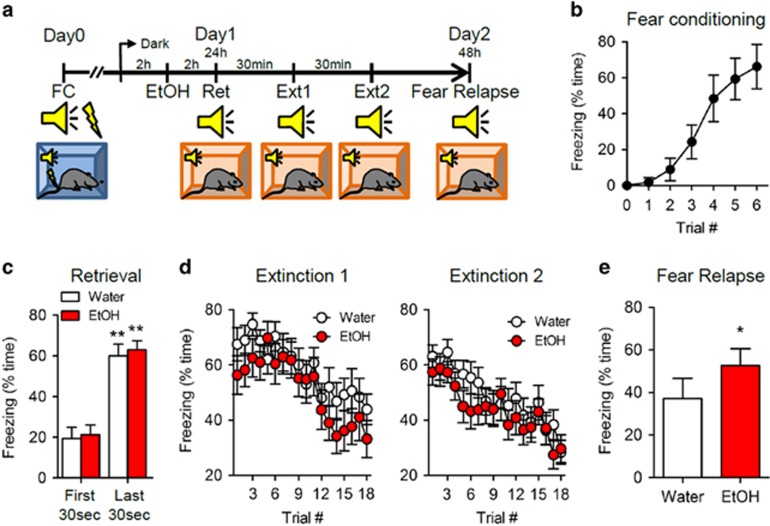
EtOH interferes with the consolidation of behavioral extinction. (**a**) Schematic illustration of the fear conditioning paradigm. (**b**) Freezing (% time) for each of the CS–US pairing trails during day 0 of fear conditioning, demonstrating an establishment of the CS–US pairing. (**c**) Freezing during the first and last 30 s of the retrieval period showing that EtOH did not interfere with the reconsolidation of fear conditioning. (**d**) Repeated CS exposure during extinction trials resulted in a gradual decrease in the CR that was similar in mice previously exposed to EtOH or water. (**e**) Fear relapse trials showing reinstatement of the CR in mice exposed to EtOH. Data are presented as mean±s.e.m. of *n*=20 per condition; significant differences between first 30 s and last 30 s in retrieval; between water and EtOH were determined by one-way ANOVA. *P<0.05, **P<0.01 compared with water. ANOVA, analysis of variance; CR, conditioned response; CS, conditioned stimulus; EtOH, ethanol; FC, fear conditioning; US, unconditioned stimulus.

We first determined whether the self-administration of EtOH after auditory fear conditioning interfered with extinction training using an ABB model in which conditioning was conducted in context A, followed by extinction, and fear relapse testing in context B. Fear conditioning was established using an auditory CS paired with foot shock (US; Figure 1a). Establishment of CS–US behavior was demonstrated by a linear increase, and plateau in freezing behavior over six paired CS–US presentations (Figure 1b). Twenty-four hours following establishment of auditory fear conditioning, mice were given free access to 20% EtOH for 2 h, beginning 2 h into their dark cycle. This method of EtOH self-administration produces spontaneous binge intake without prior training.^7, 8^ EtOH intake was slightly higher than water intake over this time period, and produced a blood EtOH concentration of 9.6±1.1 ng μl^−1^ after 2 h of EtOH drinking (Supplementary Figure 1a, b). Two hours after drinking, we introduced an extinction protocol of reconsolidation update previously shown to permanently attenuate fear memory.^9, 12^ The CS was presented once to retrieve a consolidated memory that is labile to modification for several hours after its retrieval.^13^ Freezing behavior during the last 30 s of this retrieval cue was similar to freezing recorded during the last trials of CS–US training, suggesting that EtOH did not impair the consolidation, or retrieval of auditory fear conditioning (Figure 1c). The retrieval cue was followed by extinction training (two rounds of 18 trails each), in which the CS was presented without the US. The extinction protocol resulted in a progressive reduction of freezing behavior that was similar in both water and EtOH groups (blood EtOH was 7.9±1.1 ng μl^−1^; Supplementary Figure 1b), suggesting that EtOH did not interfere with extinction training (Figure 1d). However, EtOH-exposed mice exhibited renewal of conditioned fear evidenced by increased freezing behavior during fear relapse tests (Figure 1e; blood EtOH was undetectable at this time point; Supplementary Figure 1b). We interpret these findings to suggest that the binge drinking of EtOH before extinction produced an internal context that was different from the internal state of the animal during fear relapse testing. These conflicting interoceptive cues impaired behavioral extinction.

### Failure to consolidate behavioral extinction following EtOH binge drinking is associated with modifications in the phosphorylation of hippocampal GluR1

**Figure 2:**
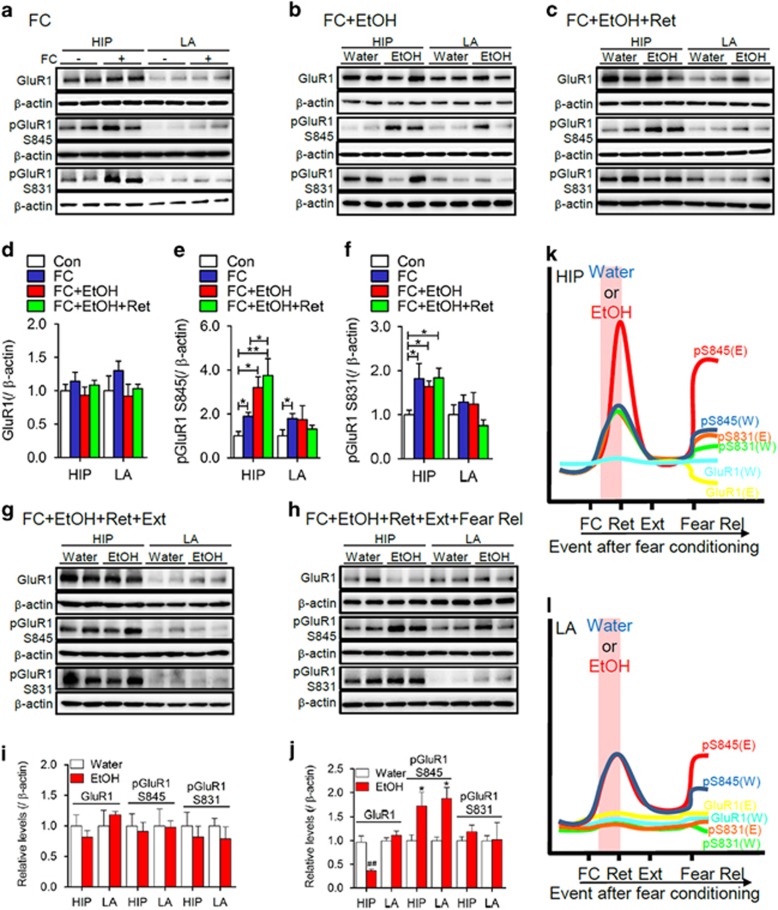
EtOH enhances phosphorylation of GluR1 at serine 845 in hippocampus. Representative immunoblots of GluR1, GluR1S845, GluR1S831 and quantitative analysis of band density from HIP and LA following the indicated treatment conditions. (**a**, **d**) Twenty-four hours after FC (lanes indicated+are fear conditioned, and lanes indicated− are sham control). (**b**,**e**) Twenty-four hours after FC including 2 h of exposure to binge EtOH intake (ETOH) or water. (**c**,**f**) Twenty-four hours after FC including 2 h of exposure to binge EtOH intake or water, and following the retrieval cue (Ret; tone). (**g**,**i**) Immediately following extinction for the indicated treatment groups. (**h**, **j**) Twenty-four hours following extinction and immediately following the fear relapse test. Representative traces show relative changes in protein expression of GluR1, GluR1S831 and GluR1S845 for the indicated treatment conditions in HIP (**k**) and LA (**l**). Data are mean±s.d. *P<0.05, **P<0.01 compared with the indicated treatment condition. EtOH, ethanol; FC, fear conditioning; HIP, hippocampus; LA, lateral amygdala.

We next sought to determine the molecular mechanisms by which EtOH impairs behavioral extinction. Fear conditioning and its maintenance are associated with a potentiation of glutamatergic synaptic transmission that involves modifications in the surface trafficking of AMPA receptors (AMPARs).^5, 9^ Surface localization of AMPARs is regulated by protein–protein interactions, posttranslational modifications and transient alterations in the lipid composition of plasma membranes.^14, 15^ As EtOH is known to modify kinase and phosphatase activity,^16, 17^ and the fluidity of lipid bilayers,^18^ we reasoned that failure to consolidate behavioral extinction following self-administration of EtOH may be related to modification of AMPAR expression and/or plasma membrane trafficking. We used GluR1 phosphorylated on serine 845 and serine 831 as surrogate measures for surface localization of AMPAR in brain tissue homogenates,^15^ from animals exposed to fear conditioning and extinction. Fear conditioning did not modify levels of total GluR1 in the lateral amygdala or hippocampus (Figures 2a and d), but resulted in increased levels of pGluR1S845 in the hippocampus and lateral amygdala (Figures 2a and e), and increased pGluR1S831 in the hippocampus (Figures 2a and f). Binge EtOH intake after fear conditioning did not alter total GluR1 in the lateral amygdala or hippocampus (Figures 2b and d), increased pGluR1S845 in the hippocampus and lateral amygdala (Figures 2b and e) and selectively increased pGluR1S831 in the hippocampus (Figures 2b and f). Following the retrieval cue, there was a further and selective increase of pGluR1S845 in the hippocampus (Figures 2c and e). These data demonstrate that the phosphorylation of GluR1 in hippocampus and amygdala during fear conditioning is modified by EtOH, and that retrieval of the fear association is accompanied by a selective increase in the phosphorylation of GluR1S845 in hippocampus (Figures 2k and l). Following extinction training, levels of total GluR1, pGluR1S831 and pGluR1S835 were similar in control and binge EtOH-exposed mice (Figures 2g and i). However, the next day following fear relapse testing, mice previously exposed to binge EtOH consumption exhibited reduced levels of total GluR1 and increased levels of pGluR1S845 in the hippocampus compared with control mice (Figures 2h and j). Thus, a failure to consolidate the behavioral extinction of fear memory in mice exposed to binge EtOH consumption was associated with a hippocampal-specific GluR1 phosphorylation known to regulate the surface expression of AMPARs (Figures 2k and l). A single binge exposure to EtOH was not sufficient to alter synaptic density in the medial prefrontal cortex (Supplementary Figure 2a, b), as has been previously reported following chronic intermittent EtOH.^19^ These data suggest that EtOH modifies the internal state of the animal, and that these interoceptive cues are processed through the hippocampus by mechanisms that involve modifications in the surface expression of AMPAR.

### EtOH modifies plasma membrane structure and redistributes GluR1 to membrane microdomains

**Figure 3:**
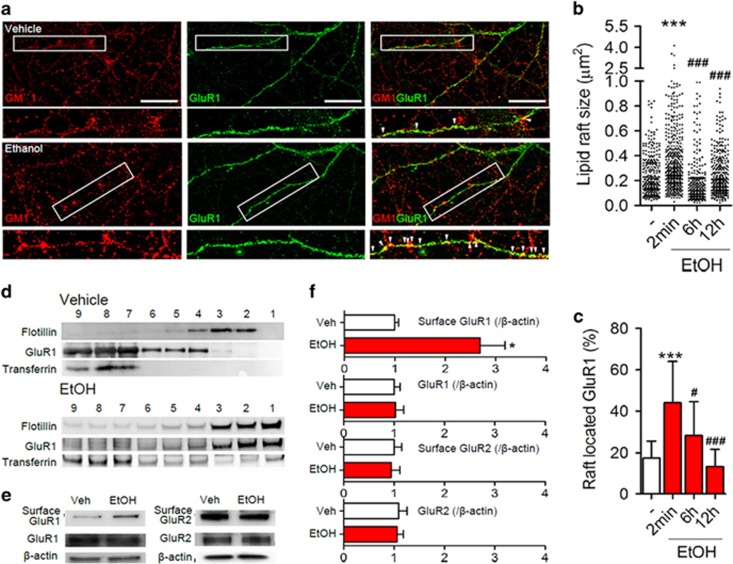
EtOH modifies the biophysical properties of cellular membranes and redistributes GluR1 to microdomains. (**a**) Representative immunofluorescent images showing EtOH facilitated a rapid (2 min) redistribution of the microdomains (GM1+, red) into enlarged clusters along dendrites, simultaneously resulted in redistributions of GluR1 (green) with clusters located to GM1+ microdomains (arrowheads indicate GM1/GluR1-co-localized microdomains). (**b**) Size of individual GM1+ microdomains, and (**c**) number of GluR1 located to GM1+ microdomains (*n*=276–440 microdomains from a minimum of 21 dendrites and 3 separate cultures per condition). (**d**) Representative immunoblots showing detergent-resistant membrane microdomains isolated by density centrifugation containing a membrane microdomain-enriched protein Flotillin (largely located to the more buoyant lipid-rich fractions, 1–3), a non-microdomain protein Transferrin (largely located outside of membrane microdomains, fractions 7–9) and GluR1. EtOH (2 min) induced a redistribution of GluR1 to membrane microdomains. (**e**) Representative immunoblots of surface biotin protein labeling and immunoprecipitation with subsequent immunoblotting for GluR1 or GluR2. (**f**) Quantitative analysis of immunoblots showing that EtOH specifically increased surface GluR1 but not total GluR1. Data are presented as mean±s.d. EtOH, ethanol. *P<0.05, ***P<0.001 compared with vehicle; ^#^*P*<0.05, ^###^*P*<0.001 compared with EtOH. Scale bar, 20 μm.

We next determined whether EtOH modified plasma membrane structure and localization of GluR1 in primary hippocampal neurons. Membrane microdomains were identified using CTX555, a fluorescent-conjugated inactive cholera toxin subunit B that preferentially binds the lipid raft-enriched ganglioside GM1.^20^ EtOH rapidly (within 2 min) re-distributed GM1 along neuronal dendrites into clusters that were 1.9-fold larger compared with GM1+ domains in vehicle-treated control cultures (Figures 3a and b). GM1+ clusters in EtOH-treated neurons gradually dispersed and were similar to control within 6 h (Figure 3b). As AMPARs are known to be localized to regions of synaptic membranes enriched with GM1,^21^ we next determined whether EtOH modified the distribution of GluR1. In control cultures, 19.0±7.6% of GluR1 was located into GM1+ regions and often appeared as small clusters. A 2 min exposure of neurons to EtOH increased GM1 localized GluR1 to 44.2±17.4% (2.3-fold increase; Figures 3a and c). The amount of GluR1 localized with GM1 returned to control levels within 6 h of EtOH treatment (Figure 3c). We confirmed that EtOH treatment promoted a redistribution of GluR1 to lipid rafts using density centrifugation to isolate a detergent-resistant membrane fraction. In control conditions, GluR1 was primarily distributed to non-lipid raft fractions identified by transferrin, with a smaller portion of GluR1 localized to more buoyant fractions identified by flotillin, a protein known to localize to lipid rafts (Figure 3d). EtOH exposure promoted a rapid redistribution of GluR1 to lipid raft fractions (Figure 3d). We next used biotin-labeling of surface proteins and immunoblotting to determine whether EtOH modified the surface expression of GluR1 and GluR2. EtOH did not alter total levels of GluR1 or GluR2, but did increase surface expression of GluR1 by 2.7-fold without altering surface expression of the calcium-impermeable AMPAR subunit GluR2 (Figures 3e and f). These data suggest that EtOH redistributes CP-AMPARs into stabilized GM1+ membrane microdomains.

### AMPA-evoked calcium influx is amplified in membrane microdomains

**Figure 4:**
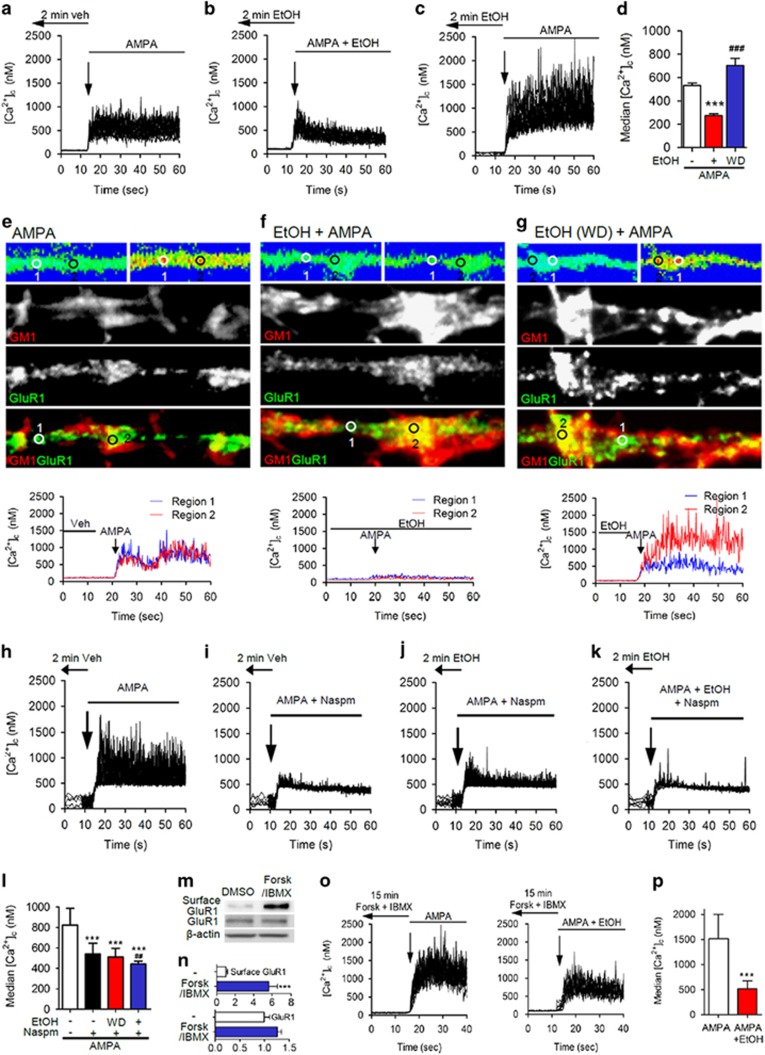
Sensitization of focal AMPA-evoked calcium responses in membrane microdomains. AMPA-evoked calcium transients were measured along dendritic branches using the ratiometric calcium probe Fura-2 at the rate of 10 image pairs per second. Focal calcium bursts evoked by (**a**) AMPA (20 μM) were (**b**) suppressed when EtOH remained present in the bathing media, and (**c**) enhanced during EtOH WD. (**d**) Quantitation of AMPA-evoked calcium transients showing the median amplitudes of calcium responses for the indicated conditions. (**e–g**) Representative images for the indicated conditions showing (from top to bottom) pseudocolor images of baseline and AMPA-evoked calcium transients, immunofluorescent staining of the same dendrite for GM1, GluR1 and the merged images. Lower tracings show baseline and AMPA-evoked calcium responses for the indicated regions. (**h–k**) Representative traces of AMPA-evoked calcium transients evoked after a 2 min pre-exposure to vehicle or EtOH were inhibited by the selective calcium-permeable AMPAR antagonist, Naspm trihydrochloride (Naspm, 50 μM). (**l**) Quantification of AMPA-evoked calcium transients showing the median amplitudes of calcium currents evoked under the indicated conditions. (**m**) Representative immunoblots of rat hippocampal neurons treated with forskolin (Forsk, 20 μM) and IBMX (50 μM). (**n**) Quantification of surface GluR1 and total GluR1 after treatment of forskolin and IBMX. (**o**) Representative traces of AMPA-evoked calcium responses in neurons pretreated with forskolin and IBMX and then continuously exposed to EtOH. (**p**) Quantification of AMPA-evoked calcium responses for the indicated conditions. Data are median±s.d. ***P<0.001; *^##^P*<0.01; *^###^P*<0.001. AMPA, n-2-amino-3-(5-methyl-3-oxo-1,2-oxazol-4-yl) propanoic acid; AMPAR, AMPA receptor; EtOH, ethanol; IBMX, 3-isobutyl-1-methylxanthine; WD, withdrawal.

We next determined whether EtOH modified focal AMPA-evoked calcium bursts acquired at the rate of 10 images per second in multiple 1 μm diameter regions along the length of dendritic branches. Perfusion of neurons with AMPA evoked calcium bursts with average amplitudes of 532.1±116.7 nM that did not desensitize during 60 s of continuous AMPA treatment (Figures 4a and d). A 2 min pretreatment with EtOH followed by continuous EtOH exposure during AMPA treatment produced a rapid desensitization of calcium responses with average amplitudes of 273.3±91.0 nM (Figures 4b and d). Surprisingly, when we pre-exposed neurons to EtOH for 2 min and then perfused cells with AMPA during EtOH washout, calcium bursts were enhanced with average amplitudes of 702.7±196.5 nM (Figures 4c and d). Labeling neurons with CTX555 to identify GM1+ microdomains and immunostaining for GluR1 immediately following calcium measurements revealed that focal AMPA-evoked calcium bursts were similar when GluR1 was located inside or outside of GM1+ membrane microdomains in control conditions, and were similarly blunted in the presence of EtOH (Figures 4e and f). In contrast, AMPA-evoked calcium responses were enhanced only in GM1+ membrane microdomains during EtOH washout (Figure 4g). Although we conducted AMPA-evoked calcium response experiments in the presence of antagonists to NMDA receptors, voltage-operated calcium channels and sodium channels, we confirmed that EtOH modified the response of CP-AMPARs using Naspm trihydrochloride (Naspm), a selective CP-AMPAR inhibitor. AMPA-evoked calcium responses were generally diminished in the presence of Naspm compared with untreated control cultures, and EtOH did not modify the amplitude of AMPA-evoked calcium responses in the presence of Naspm (Figures 4h–l). These findings suggest that EtOH promotes an increase in the surface expression of CP-AMPARs, and blocks calcium conductance through these channels. During EtOH washout, surface-located CP-AMPARs remained clustered in GM1+ membrane microdomains where calcium bursts are enhanced. To confirm that EtOH blocked calcium conductance, we artificially induced surface expression of GluR1 by treatment with forskolin to activate cAMP and PKA-mediated phosphorylation of GluR1,^22^ and measured AMPA-evoked calcium bursts in neuronal dendrites. Forskolin treatment rapidly increased surface expression of GluR1 5.7-fold without altering total GluR1 (Figures 4m and n), and enhanced AMPA-evoked calcium bursts from baseline in control conditions to 1514.7±485.8 nM (Figures 4o and p) in forskolin-treated cultures. Acute exposure of neurons to EtOH following forskolin treatment reduced AMPA-evoked calcium bursts to 516.8±160.3 nM (Figures 4o and p). These results demonstrate that agonist-evoked activity of CP-AMPARs is blunted in the presence of EtOH. The removal of EtOH results in a preferential increase in activity for CP-AMPARs clustered into GM1+ membrane microdomains.

### EtOH promotes PKC- and PKA-mediated phosphorylations of GluR1

Surface expression of AMPARs is regulated through PKA- and PKC-mediated phosphorylation of AMPAR subunits.^15^ We found that EtOH increased surface localization of GluR1 by 1.6-fold as determined by biotin-labeling of surface proteins followed by immunoblot (Supplementary Figure 3a, b). This increase in surface-located GluR1 was accompanied by a similar 1.5-fold increase in the phosphorylation of GluR1 on serine 845 and 831 (Supplementary Figure 3a, b). Inhibition of PKA resulted in a 22% reduction in total surface expression, a 58% reduction in phosphorylation of GluR1S845 and no effect on GluR1S831 (Supplementary Figure 3a, b). Inhibition of PKC resulted in a 72% reduction in surface expression of total GluR1, with no effect on GluR1S845 and a 69% reduction in the phosphorylation of GluR1S831 (Supplementary Figure 3a, b). Total GluR1 was not affected by EtOH, or by inhibition of PKA, or PKC (Supplementary Figure 3a, b). These data suggest that EtOH regulates the activity or accessibility of GluR1 to PKC- and PKA-mediated phosphorylation, and increases the surface localization of AMPARs.

### AMPAR antagonists rescue the impairment in extinction

**Figure 5:**
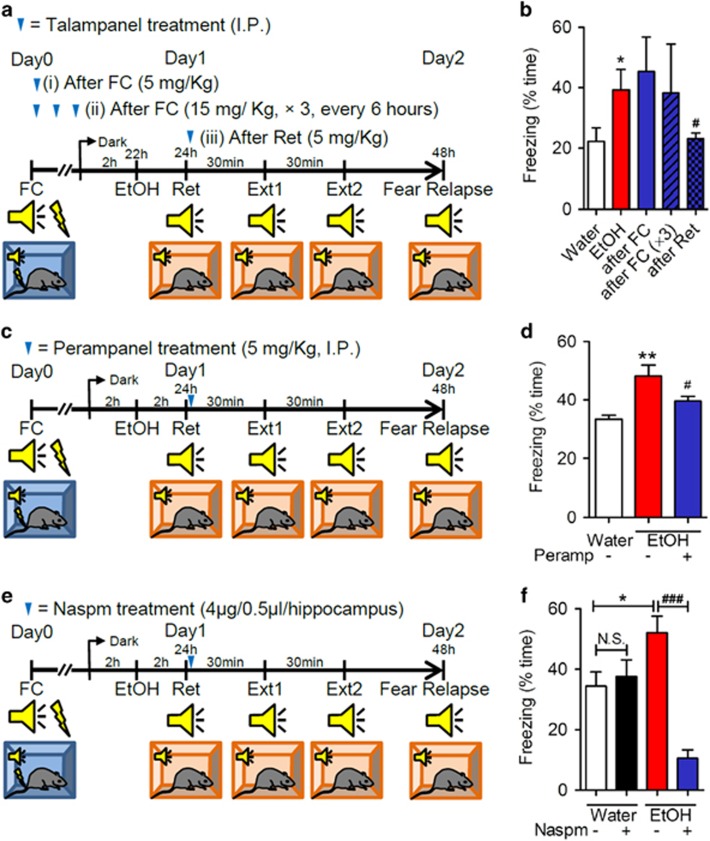
AMPAR antagonists rescue EtOH-associated impairment in the consolidation of extinction. (**a**) Schematic illustration of therapeutic time windows for Talampanel administration. Talampanel was intraperitoneally injected immediately following fear conditioning (i, 5 mg kg^−1^), three times over 18 h (ii, 6 h interval, 5 mg kg^−1^), or a single dose after retrieval (iii, 5 mg kg^−1^). Blue arrows depict dosing time and intervals. (**b**) Quantitative data showing freezing (% time) during fear relapse testing for the indicated treatment conditions. (**c**) Schematic illustration of timing for Peramapanel treatment. A single dose of Perampanel was intraperitoneally injected after retrieval (blue arrow, 5 mg kg^−1^). (**d**) Quantitative analysis of freezing behavior (% time) for the indicated treatment conditions. (**e**) Schematic illustration of timing for Naspm treatment. Mice received bilateral injections of Naspm into hippocampi (4 μg per 0.5 μl per min) after exposure to the retrieval cue. (**f**) Quantitative analysis of freezing behavior (% time) for the indicated treatment conditions. Data are mean±s.d. of *n*=10–14 animals per condition. *P<0.05, **P<0.01, ^#^*P*<0.05 and ^###^*P*<0.001. ANOVA with Tukey *post hoc* comparisons. ANOVA, analysis of variance; AMPA, n-2-amino-3-(5-methyl-3-oxo-1,2-oxazol-4-yl) propanoic acid; AMPAR, AMPA receptor; EtOH, ethanol; FC, fear conditioning.

Our *in vivo* and tissue culture findings suggest that a failure to consolidate behavioral extinction following binge drinking of EtOH is associated with alterations in neural membranes that trap CP-AMPARs at the cell surface. On the basis of previous reports that synaptic removal of CP-AMPARs is required for behavioral extinction,^9^ we reasoned that blocking AMPARs may facilitate behavioral extinction. To test this hypothesis we first administered a single dose (5 mg kg^−1^), or three doses of the non-competitive AMPAR antagonist Talampanel (6 h interval, 15 mg kg^−1^) after fear conditioning, but before binge EtOH intake (Figure 5a). The next day, a retrieval cue was followed by extinction training, and by a fear relapse test 24 h later. Treatment with Talampanel before binge drinking (single or multiple doses) did not rescue impairments in behavioral extinction, as evidenced by freezing behavior that was similar to drug-naive mice exposed to binge EtOH intake (Figure 5b). As pGluR1S845 was greatest in mice exposed to EtOH following the retrieval cue, we next administered a single dose of Talampanel (5 mg kg^−1^) following the retrieval cue. This timing of drug administration rescued behavioral extinction in mice following binge EtOH intake as demonstrated by freezing behavior in the fear relapse test that was similar to water-fed mice (Figure 5b). The dose of Talampanel used in these studies did not have an impact on locomotor activity (Supplementary Figure 4), which can occur at higher doses of Talampanel.^23^ A similar effect to rescue behavioral extinction in mice following binge EtOH drinking was produced with Perampanel (2-(2-oxo-1-phenyl-5-pyridin-2-yl-1,2 dihydropyridin-3-yl) benzonitrile hydrate (4:3; 5 mg kg^−1^, i.p.; Figures 5c and d), a second-generation non-competitive AMPAR antagonist.^24^ Finally, to demonstrate that CP-AMPARs in the hippocampus were critical to process interoceptive context, we directly infused Naspm into the CA1 region of the hippocampus immediately following the retrieval cue in mice pre-exposed to binge EtOH (Figure 5e, Supplementary Figure 5). This procedure restored extinction in mice pre-exposed to EtOH, but had no effect on extinction in water-fed mice (Figure 5f).

## Discussion

Fear conditioning and the extinction of conditioned fear involves alterations in the strength of neural circuits that connect the amygdala, prefrontal cortex and hippocampus. Conditioning-induced plasticity of the basolateral amygdala precedes, and is thought to drive the conditioned fear response.^25, 26^ Prefrontal cortical regions have critical roles in the expression of conditioned fear,^27^ and extinction,^28^ through competing circuits that connect the prefrontal regions to the basolateral amygdala and prelimbic cortex, respectively. The hippocampus encodes contextual aspects of conditioned fear, and has major projections to both the prefrontal cortex and the basolateral amygdala.^29^ Contextual aspects of fear conditioning and extinction are thought to include the internal state of the organism; however, roles for this interoception in fear conditioning or extinction have not been experimentally tested. Our findings demonstrate that EtOH interferes with extinction by modifying the internal context of extinction training. Temporal modifications in the phosphorylation of GluR1 during fear conditioning and extinction suggested that changes in the surface expression of CP-AMPARs underlie this behavioral phenomenon. Mechanistic studies supported this notion, demonstrating that EtOH rapidly modifies the biophysical structure of neuronal membranes with a collapse of GM1 into microdomains enriched for surface-located CP-AMPARs. AMPA-evoked calcium responses were blocked in the presence of EtOH, and enhanced during washout of EtOH, suggesting that EtOH also directly interacted with AMPARs.

Extinction of the conditioned fear response is largely regarded as a new learning event that results in the formation of an extinction network that inhibits the fear network.^30^ The effects of extinction are highly dependent on the hippocampal encoding of context,^31^ and are more durable when extinction is conducted in a context that is novel from the conditioning context.^5^ When fear relapse testing is conducted in the same context as extinction, the hippocampus drives prefrontal activation of an extinction circuit in the amygdala that suppresses the expression of fear.^32, 33^ When the context of fear relapse testing is novel from the extinction context, prefrontal activation is reduced, and the hippocampus is thought to enhance activity of a fear circuit in amygdala, leading to renewed fear.^2, 34, 35^ Chronic intermittent EtOH exposure produces reductions in infralimbic prefrontal cortical dendritic density, and declines in NMDAR-mediated currents that are associated with impairments in extinction,^36^ suggesting that chronic EtOH interferes with the ability of hippocampus to drive prefrontal circuitry that normally suppress fear when the extinction and fear relapse testing contexts are identical. In this study we used a single round of EtOH self-administration to determine whether EtOH modified the hippocampal encoding of context. In our paradigm, extinction and fear relapse testing were conducted in the same environmental context, and the only experimental difference was in the internal state of the animal at the time of extinction training (2 h after EtOH or water intake). This paradigm did not impair the process of extinction, but interfered with extinction as evidenced by fear renewal (freezing) during fear relapse trials. We interpret these findings to suggest that the internal state of an animal at the time of extinction influences the perception of context. Biochemical studies of brain tissues from these animals, and mechanistic studies in cultured neurons implicated hippocampal AMPARs in this behavioral phenomenon.

Extinguishment of conditioned fear involves the retrieval (reconsolidation) of a fear memory that is liable to modification for several hours after retrieval.^13^ This process is thought to allow for the incorporation of new information into an updated memory trace.^37^ During memory reconsolidation, calcium-impermeable AMPARs in the amygdala are replaced by CP-AMPARs that internalize during the process of extinction.^9, 38^ We observed a similar pattern of phosphorylation on GluR1S845 and GluR1S831 in the amygdala of animals in this study. We also found that this pattern GluR1 phosphorylation was mirrored in the hippocampus. The self-administration of binge EtOH intake 2 h before extinction training produced a robust and selective increase in the phosphorylation of GluR1S45 only in the hippocampus. We recapitulated this effect in cultured hippocampal neurons, and observed that EtOH induced a rapid re-organization of the plasma membrane, with clustering of GluR1S845 into GM1+ membrane microdomains. AMPA-evoked calcium flux was inhibited in the presence of EtOH, and exaggerated during the washout of EtOH. These findings suggest that EtOH modified the timing of CP-AMPAR internalization, and that this perturbation interfered with permanence of extinction encoding. Indeed, blockade of AMPARs with talampanel or parampanel restored the permanence of extinction, but only when the drug was given after the retrieval cue and before extinction training.

These findings suggest that cognitive/behavioral desensitization therapies for the treatment of PTSD in humans could be complicated by EtOH use, and potentially by other prescribed and/or illicit substances that modify internal state and perception. PTSD can occur following traumatic life events such as combat exposure, abuse, a natural disaster or serious accident that involves threat of injury or death. Typical PTSD symptoms include flashbacks, affective disorders, hyperarousal and avoidance of situations and/or people that remind the individual of the traumatic event. Cognitive-behavioral therapies including eye movement desensitization and reprocessing therapies, cognitive approaches and desensitization treatments attempt to modify the association of these trigger events with the feelings of arousal and fear. The positive effects of psychotherapy can be circumvented by binge alcohol abuse,^39, 40, 41^ which is more common in individuals with PTSD compared with the general population (60–80% of PTSD patients).^42, 43, 44^ It may be possible to improve the efficiency of desensitization through the use of selective AMPAR antagonists provided during critical intervals of desensitization therapy.
