# Supplementary figures and images for: Hippocampal encoding of interoceptive context during fear conditioning
Source: Transl Psychiatry. 2017 Jan 3;7(1):e991–. doi: 10.1038/tp.2016.254 (PMC5545722; doi:10.1038/tp.2016.254)

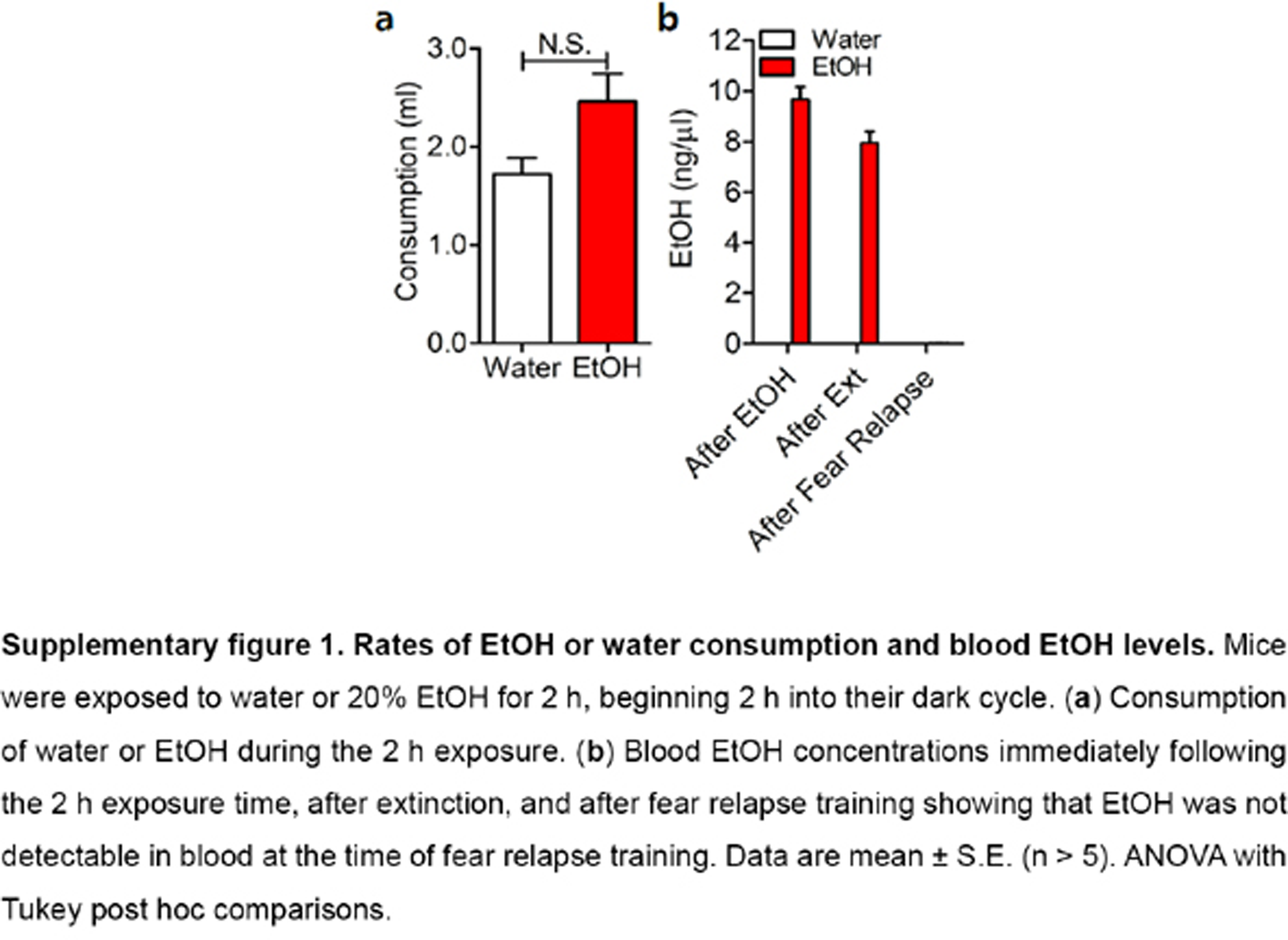

Supplement: Supplementary Figure 1 [file tp2016254x1.tif]

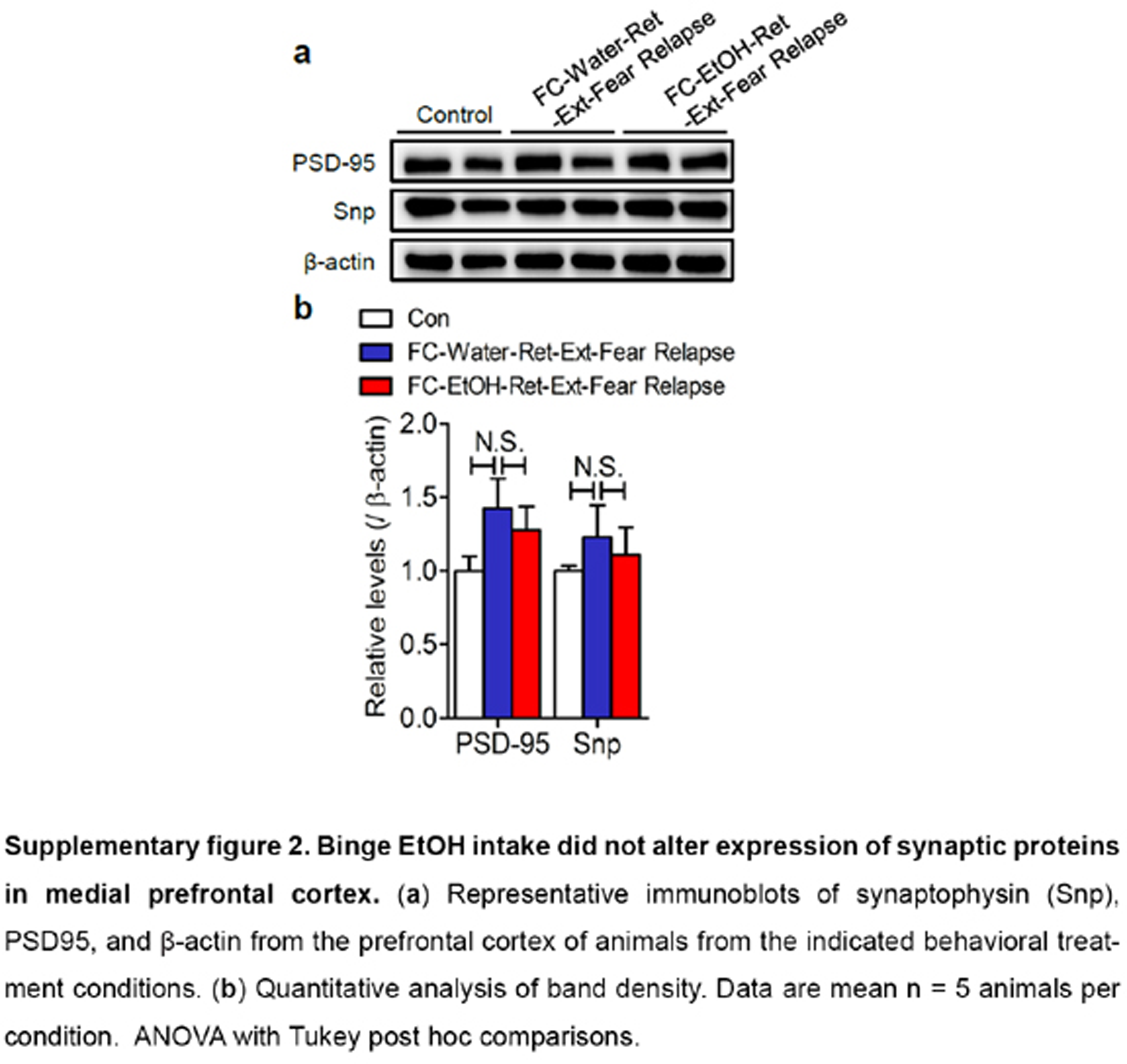

Supplement: Supplementary Figure 2 [file tp2016254x2.tif]

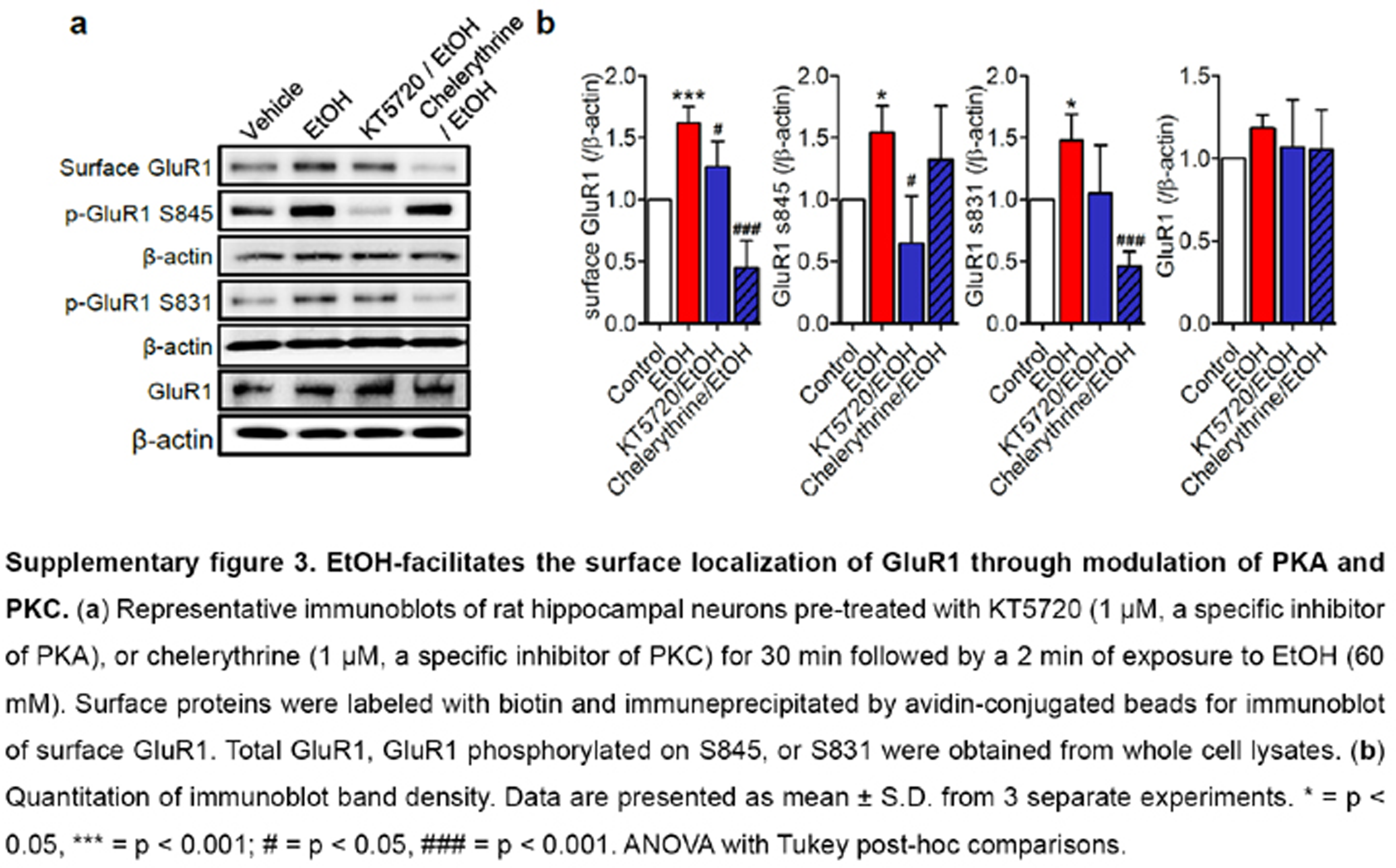

Supplement: Supplementary Figure 3 [file tp2016254x3.tif]

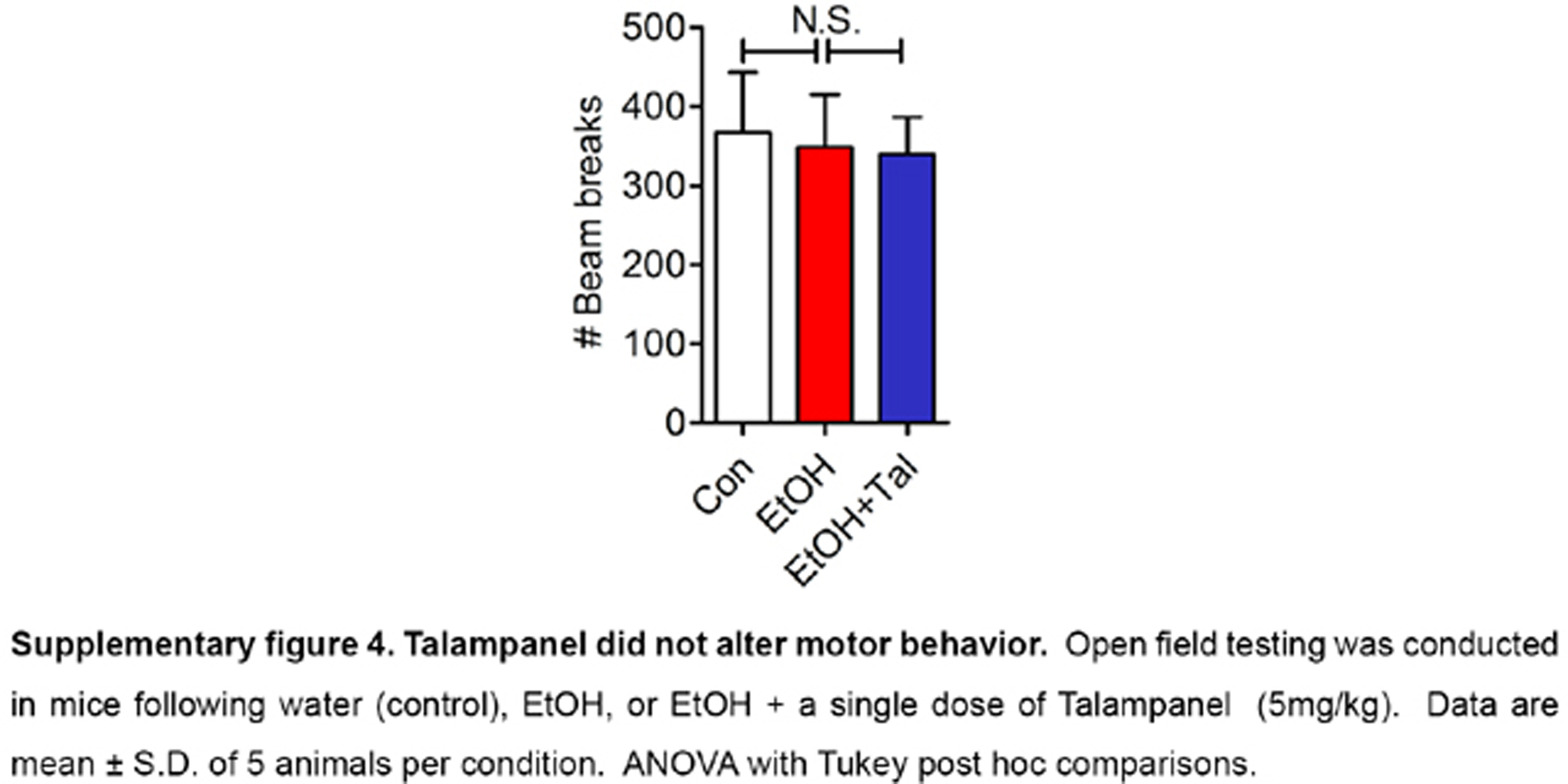

Supplement: Supplementary Figure 4 [file tp2016254x4.tif]

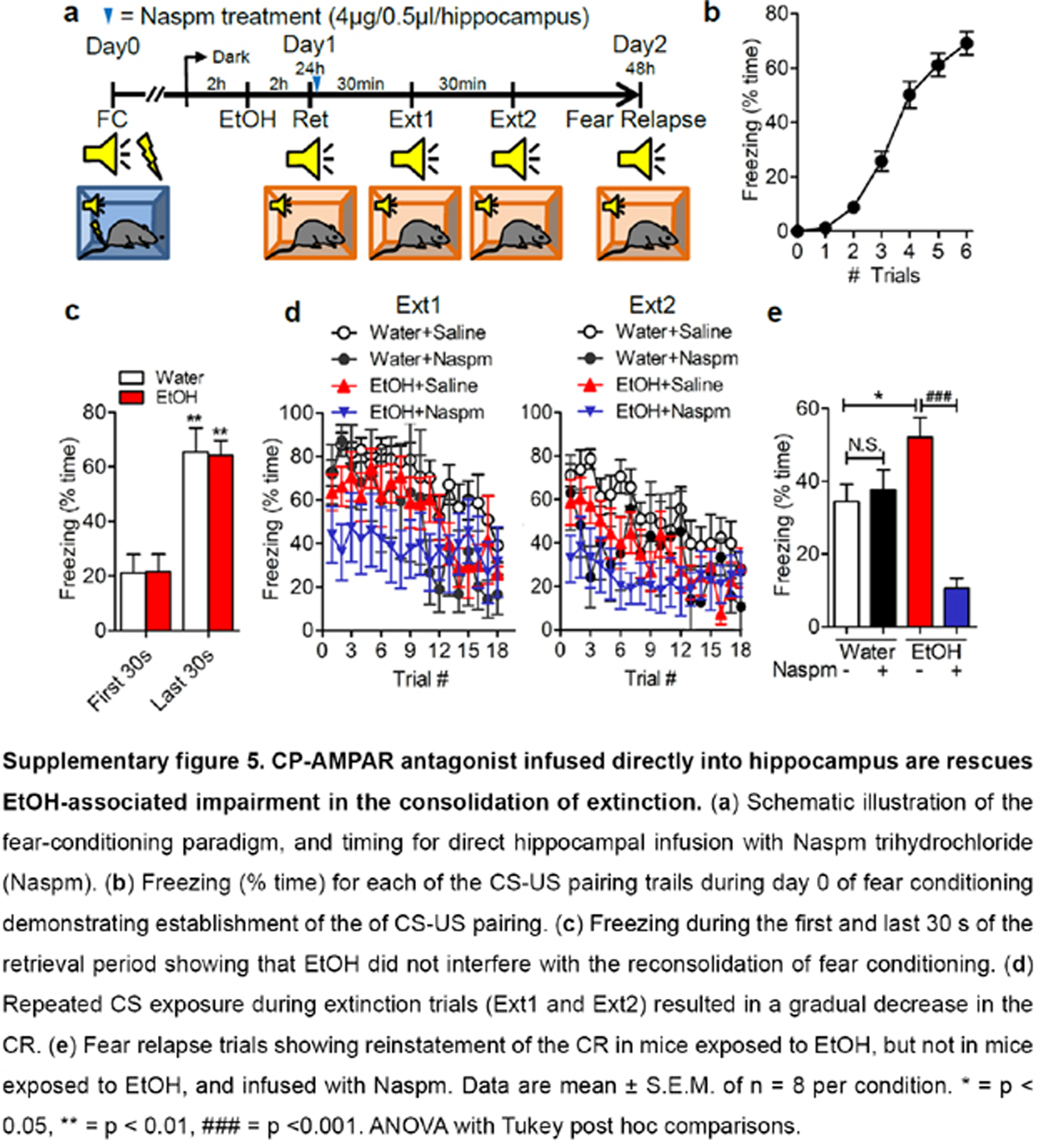

Supplement: Supplementary Figure 5 [file tp2016254x5.tif]
